# "The Hospital" Medical Book Supplement.—No. 3

**Published:** 1908-01-18

**Authors:** 


### "THE HOSPITAL" MEDICAL BOOK SUPPLEMENT.—No. III.

CONTENTS.

**Stray Notes—**
Our Objects—Co operate, Doctor—A Change—The Medical Directory—Who's Who—Merck's Index for 1907—A Useful Pocket Book - 427
**Surgery—**
The Treatment of Syphilis - 429
Diseases of the Ear - 430
Surgical Pathology and Morbid Anatomy - 430
**Medicine—**
The Standard Family Physician - 428
The Borderland of Epilepsy - 429
The Reduction of Cancer - 429
Some Successful Prescriptions - 429
**Special Subjects—**
The Ophthalmoscope: A Manual for Students - 430
Aids to Pathology - 430

### STRAY NOTES.

**Our Objects.**

IN former issues of THE HOSPITAL we have already alluded to the ideal which we have set before us in organising this literary supplement. It is permissible, however, to repeat what our objects and aims are, so as to keep them before our readers. We are endeavouring to make this book page of real interest to the practising physician: to give, in as condensed a form as is compatible with lucidity, a review of such books as appeal more especially to the general practitioner. The attempt to weigh and discriminate is a difficult one; the task of choosing and rejecting between a number of good books is often an impossible one for the busy doctor who has no time to make his selection himself. We have no desire to interfere with individual choice, for in the selection of books, as in most things, individual taste and preferences must always be the predominating factors. What we can do however, and what we hope to effect, is to help in the task of selection by eliminating from the catalogue set before the reader of book reviews such volumes as are obviously unsuited for the general practioner's library, and laying stress upon the merits of such as we consider worthy of the doctor's consideration.

**Co-operate, Doctor.**

IN this task we desire the help and co-operation of our readers, individually and collectively. You, doctor, who are a general practitioner, you ought to know exactly what you want. Perhaps you have not attempted to catalogue your wants; do so now. When you have a moment to spare, criticise us frankly and freely. Let us know in what you consider this paper deficient, how you think it may be improved, in what manner you deem it can best achieve the main object we have in view, to make THE HOSPITAL the ideal general practitioner's paper in this country. All your suggestions will receive careful and grateful consideration, and the fact that you have made them will come as encouragement to us. Bear in mind that the future of the paper is very much in your hands. Unless we know definitely your wants, we can only work in the twilight. Co-operate, take a personal share, as it were, in your weekly paper, and you will soon realise how very effectively you can aid towards improvement and success.

---

**A Change.**

MR. YOUNG J. PENTLAND, of Edinburgh, has relinquished his publishing business in favour of Mr. Henry Frowde, Oxford University Press, and Messrs. Hodder and Stoughton. The copyright volumes transferred include the well-known "Text-book and Manual of Anatomy," by Professor D. J. Cunningham; the "Text-book of Physiology," by Professor Schafer; the "Manual of Bacteriology," by Professors Muir and Ritchie; "the "Manual of Surgery," by Messrs. Thomson and Miles; the "Outlines of Zoology," by Professor J. Arthur Thomson, etc. These important works will for the future be published by Mr. Frowde and Messrs. Hodder and Stoughton, the publishers of the excellent Oxford Medical Manuals.

**The Medical Directory.**

THE sixty-fourth annual issue of "The Medical Directory" (J. and A. Churchill, 7 Great Marlborough Street, 14s. net), made its appearance, with praiseworthy punctuality, early in December last. As usual, it is an excellent reference book. Mr. Armstrong's revision of Mr. Glenn's original article on "Laws Affecting the Medical Profession is fully up-to-date, and contains much information. Every newly-qualified practitioner should make himself thoroughly acquainted with these abstracts, particularly with those dealing with recent legislation, such as, for instance, the Registration of Births and Deaths, the Notification of Births Act, 1907, and the Workmen's Compensation Act, 1906. The medical summary gives a net increase of 338 names added to the directory during the year, with a net total of 39,703 names tabulated. Many of the notes given under specific names are old summaries, and many practitioners have merely returned their names, qualifications, and addresses. There is much to be said in favour of this modest manner of doing things.

**Who's Who.**

THIS is another excellent reference book, published by A. and C. Black, Soho Square, at 10s. 6d. net. It is frankly and honestly an advertising directory, but one which we should not like to do without. A veritable mine of information and an interesting book to peruse, if only to see how notabilities spend their time in recreation. The majority of medical men who have achieved the distinction of figuring on these hoardings play golf. One swims, and one frankly avows his preference for motoring. All biographies are excellently done, with condensation carried to a fine art.

**Merck's Index for 1907.**

THE third edition of this useful compilation of drugs and pharmaceutical products has just been published. It is an encyclopædia for the chemist, pharmacist, and physician, and as a reference volume it should have a corner to itself on the library shelves. Comparative values of the various drugs are given, and accurate doses are listed, while in each case synonyms are also given. The index is published by Messrs. Merck and Co., 15 University Place, New York, and and can be obtained through any bookseller.

**A Useful Pocket Book.**

WE have received from Messrs. Scott and Bowne, Ltd., the well-known manufacturers of Scott's Emulsion of Cod Liver Oil, a copy of their "Doctor's Diary and Emergency Notebook." This little pocket diary contains a large amount of useful information crammed into the minimum space. The old features are included, and have been carefully revised. The diary may be obtained on application to the publishers, 10 and 11 Stonecutter Street, E.C.

### MEDICINE.

The Standard Family Physician. Edited by Sir James Crichton-Browne, M.D., LL.D., F.R.S., Lord Chancellor's Visitor in Lunacy, London; Sir William H. Broadbent, Bart., K.C.V.O., M.D., F.R.S., late Physician-in-Ordinary to the King and Prince of Wales; Alfred T. Schofield, M.D., London; Professor Karl Reissig, M.D., Hamburg; and S. E. Jelliffe, A.M., M.D., Ph.D., Columbia University, New York, U.S.A. Small 4to. In three volumes. Published by Funk and Wagnalls Company, London and New York. 1907.)

It is with considerable misgiving that we look into and read these three large volumes. The nature of our misgiving we will give presently; before we do so we will briefly outline the plan and scope of the work.

The first xcii pages are devoted to short chapters upon such subjects as "The Family Physician," and the ideal relationships that should subsist between the family and the doctor; "The Journey of Life and the Laws of Health," with a brief discussion of the effects of inheritance, the three stages of life, the degrees of healthiness, the effects of ignorance and of wilful neglect as causes of ill-health, and so forth; "Dreamy Mental States," reprinted from Sir James Crichton-Browne's Cavendish Lecture; "The Prevention of Consumption and other Forms of Tuberculosis," by the late Sir William Broadbent, a popular account of the lines upon which the laity may help in checking the inroads of the white scourge; "The Sanitary Question-box," a handy guide to the essentials of sanitation and ventilation in the home, arranged in the form of question and answer, such as "Should plants be kept in sleeping-rooms? No, they should not. Florists often suffer from malaria contracted in greenhouses, and flower-pots and window-boxes are excellent places for germs to develop." "How often should a cellar be whitewashed? . . ." "Is it dangerous to live near a cemetery? . . ." "How can one avoid the risk of cracking thin glass in opening windows to air a dining-room in very cold weather? . . ." and so forth. Then follow seventy-seven pages upon anatomy, described from a popular point of view, and yet including such out-of-the-way things as the platysma myoides, Cowper's glands, and the epoöphoron. The next twenty pages consist of General Remarks on Disease, with pictures of ringworm fungi, gonococci, and other organisms, even including so rare a hæmatozoon as the spirillum of relapsing fever.

After these introductory chapters the style changes entirely, the second half of Volume I., the whole of Volume II., and the greater part of Volume III. being devoted to diseases, drugs, various forms of treatment, and allied conditions, described in encyclopædic form in alphabetical order. The sorts of things dealt with may be gathered from the first few headlines—namely, Abdomen, Abdominal Pains, Abortion, Absinthism, Abscess, Acne, Aconite, Actinomycosis, Adder-bite, Addison's Disease, Adiposis Dolorosa, Adolescence, Agaricus, Agglutination, Agglutinins, Agorophobia, Ague, Air-bath, and so forth to the end of Z. The average length of the account of each thing is something like half a page or less, though here and there the letterpress about such headings as Alcohol, Massage, Baths, or Venereal Disease extends to several pages. The descriptions are distinctly written for the laity, and whenever possible there is a statement as to what are the indications for sending for a doctor and what procedure should be adopted in cases of emergency until the doctor arrives. The spelling, we may incidentally note, is American—sulphate is spelt sulfate; chloride, chlorid; anæmia, anemia; syrup, sirup, and so on.

The printing and almost all the illustrations are very good. They could not be so good if the paper were not a heavy one, with the result that each volume, which is a couple of inches thick, is too heavy to hold long in the hand.

---

The purpose which the editors profess to have had in mind in producing this large work was to provide a practical international encyclopædia of medicine and hygiene especially prepared for the household. The result is certainly an encyclopædia, and with equal certainty it is not likely to be of as much service to medical men as many another book would be; it is probable, therefore, that the sale of the work will be mainly amongst the public in lands where English is the prevailing language. Our own feelings are that the sale will very likely be a large one, but that it would be much better for the public if such works were not issued at all. We think that the elementary principles of how to live healthily are things that the public should know, and we agree that the public does not yet know all it might in this connection; but we think that far more good could be done by adequate and broad-minded teaching by specially selected persons in public and private schools than by encouraging people to read about these things. Even if a book upon the subject may be necessary, the contents of that book should be very different from those of the volumes before us. A comparatively small work could be made to contain all that is most useful to the public in connection with the private and public measures that are conducive to general health. The editors assert their disbelief in Pope's line: "A little learning is a dangerous thing"; but we emphatically disagree with them when the "little learning" consists of the smattering that the uninitiated can pick up from the books before us upon such subjects as the structure of the generative organs, the venereal diseases, and so forth. The editors state that "it is ignorance and self-sufficiency that are the bane of medical science and practice, and by helping to dispel these 'The Standard Family Physician' must conduce to a timely resort to skilled medical advice"; we think the work will be much more likely to increase this baneful "self-sufficiency" than to diminish it. The great majority of people get on best by never bothering about these things at all; it is those who already have a tendency to bother about them too much who are most likely to get the books and study them, and the result may well be to make such persons more unbearable than ever to their families and to their friends.

In any case we are quite sure that no ordinary layman can derive additional health by the knowledge he can obtain from this encyclopædia upon such things at actinomycosis, athetosis, blood-letting, catalepsy, dulcamara, elephantiasis, fæcal vomiting, glaucoma, and so on. We doubt if any but a medical man would be able to interpret a great many of the things in such a way that the layman would not misunderstand them altogether; and yet the information given is far too little to be of any great value to the doctor himself. We suppose that no one would seriously hold that every layman should read and master a text-book of medicine, and yet we think that the danger he would be in from doing so would be considerably less than that to which a knowledge derived from this encyclopædia might subject him. We are irresistibly reminded of the housemaid's knee story from "Three Men in a Boat," and even more so of that delightful thing about "The Browns of Walham Green," and we think that, beyond a subconscious knowledge of the general principles of living healthily, a man is best when he does not bother himself about any of these things at all.

By way of summarising our opinion of the actual contents of the work, we think they are far more than is good for the public and far less than is of use to the profession.

The Borderland of Epilepsy. By Sir William R. Gowers, M.D. (Lond.), F.R.S., F.R.C.P. 8vo. (London: J. and A. Churchill. 1907. Price 4s. 6d. net.)

This little book is very interesting and instructive, dealing as it does with subjects whose pathology is obscure, but whose frequency of incidence in general practice is great. The letterpress is a condensed form of the lectures that the author has published upon the subject in the "Lancet" and "British Medical Journal" from time to time. The kinds of things discussed are: syncopal attacks of various kinds, petit mal, migraine, alarming seizures in which a fear of impending death is prominent, neuroses of cardiac and respiratory types, tetanoid spasms, vertigo and Menière's disease, night terrors, somnambulism and narcolepsy. Numerous cases illustrative of each of these are given, and the points of analogy with, and of distinction from, epilepsy are fully discussed. The treatment of the various conditions is also debated at some length.

The Reduction of Cancer. By the Hon. Rollo Russell. Pp. 62. Not illustrated. Cap. 8vo. (London: Longmans, Green and Co. 1907. Price 1s. 6d. net.)

The gist of this little thesis is given in the following paragraph from p. 25: "We have seen that a large number of people in many parts of the world are entirely or almost entirely untouched by cancer. The invariable condition of exemption is plain living, without addiction to toxic drinks, and generally a fare of vegetable products or fruit, with little animal food. On the other hand, every community of opposite habits is badly attacked, some are even decimated. *There is no exception.*" The foodstuffs that the author seems to blame particularly as causes of cancer are tea and coffee, though he also attacks luxuries of all sorts and excesses in diet of every kind. The book is likely to convey to the lay mind the impression that the author's views are absolutely proved to be true, and this seems to us to be a pity. The theory of the author is one which should not be put aside at once, but at the same time it should not be thrust forward in the lay press in this way withou tvery careful examination of the facts first. The author's views, and particularly his cosmopolitan method of arriving at them by statistical analyses from every available country in the world, are probably already noted by the Cancer Research Fund authorities, and ample steps will doubtless be taken to test what value can be attached to them. Meanwhile one can only regret that, instead of their being expressed in a tentative way as a theory only, they are brought before the public in book form as though there could not be a shadow of doubt as to their being true.

Some Successful Prescriptions. By A. Herbert Hart, M.D. (London: John Bale, Sons, and Danielsson, Limited. 1907. Pp. 17. Price 1s.)

This publication, of copy-book size and appearance, in paper covers, contains nineteen prescriptions, with quite brief notes of the indications for each. The author states that "On Monday, August 12, 1907, after the evening surgery, I wrote the following prescriptions, which I trust will prove useful to many of my fellow-practitioners in the noble art of medicine." The prescriptions are ordinary. The letterpress is extra-ordinary—for instance, of bismuth one reads "as a microbicide it annihilates the hoards (*sic*) of pathogenic microorganisms, which turn the intestine into a huge armed camp." We think that most of those who may inadvertently have spent a shilling upon this booklet will feel that the money has been wasted.

### SURGERY.

The Treatment of Syphilis. By Professor Alfred Fournier. English translation by Dr. C. F. Marshall of the second edition. (Rebman, London and New York, 1906. Large 8vo. Pp. xv. and 275; xii. and 219. Price 21s.)

This book contains a translation of Professor Fournier's "Treatment of Syphilis," together with a number of articles and lectures by the same author on the prevention of syphilis.

There are many excellent text-books on venereal disease in the English language, but none covers the same ground as Professor Fournier's great work. Dr. C. F. Marshall is to be congratulated on the scholarly translation of a work which must always remain a classic. Professor Fournier was Ricord's house surgeon and is a Professor at the Faculty of Medicine in Paris. He has long been recognised as the most eminent teacher in all that relates to syphilis, for he has a breadth of view and a depth of knowledge which are not often found combined, while the clearness and beauty of his writings are such as only the best masters of the French school can produce. The routine treatment of syphilis in England lags far behind the method pursued in France, Germany, and Austria, and this is due in part perhaps to the want of any systematic text-book dealing with the subject. It is known, indeed, that mercury should be given in the early stages, and iodides later in the disease. But there are many who would be unable to say what are the indications for the administration of the one drug rather than the other; what are the best forms of administration; in what ways they act; and why in the case of mercury it is advisable to adopt one method or one preparation rather than another. All these points are dealt with by Professor Fournier, and have long been known by those who are interested in the subject and who can read French. Dr. Marshall has now made them plain to the English reader. The basis of Professor Fournier's teaching consists in the fact that syphilis can be cured, but that, as the disease is seen in hospitals, no attempt is made to cure it. The patients are only seen for a short time, and as soon as the syphilitic lesions from which they are suffering have disappeared, they are discharged. The ordinary treatment of syphilis, therefore, is treatment of syphilitic lesions, not the treatment of the disease itself. If mercury is given in sufficient doses for a sufficient length of time syphilis can be absolutely cured in the great majority of cases, and if the remedy be given early enough many of the later manifestations can be prevented. With a full knowledge of all that has been done by others since syphilis came to Europe in the fifteenth century, and with the personal experience of many years' practice both in the hospitals and in private, Professor Fournier is able to speak with no uncertain voice. He condemns absolutely the opportunist method, whose advocates assert that mercury only acts when the disease is active and is useless in latent syphilis; he states that this is the method of all others which produces the greatest number of patients with advanced tertiary manifestations. The part dealing with the prophylaxis of syphilis consists of fifteen chapters, some of which are of comparatively slight importance to English readers, such as the relation of wet nurses to syphilitic sucklings and the conditions under which a wet nurse should be allowed or refused to the child of a syphilitic father. Other chapters are of fundamental importance, for they are concerned with the social dangers of syphilis, and they teach what a young man of eighteen ought to know about the dangers of venereal disease. Rebman, Limited, have produced the book in a satisfactory manner. It is light, of a convenient size, clearly printed, and is provided with two indices, which, though short, are sufficient.

Diseases of the Ear. By Hunter Tod, M.A., M.B., B.C. (Cantab.), F.R.C.S. Henry Frowde, Oxford University Press, and Hodder and Stoughton, Warwick Square, E.C. (Oxford University Manuals.)

Like most of the other volumes in this excellent series, an admirable book, both for the practitioner and the student. It is short, to the point, and clear. The plates with which it is illustrated help the reader, and there is little in the text to which the most fastidious critic can take objection. We notice in each succeeding work that is issued in this series a marked improvement in general get-up. This volume, for instance, is almost wholly free from the objectionable printer's errors which were noticeable in some of the former volumes. Mr. Tod's little text-book is by no means exhaustive, but it is as full as is consistent with the limited space he allows himself, and it is certainly one of the most useful additions to the series.

Surgical Pathology and Morbid Anatomy. By Anthony A. Bowlby, C.M.G., F.R.C.S., Surgeon to St. Bartholomew's Hospital and to the Household of His Majesty the King. Edited with the assistance of Dr. F. W. Andrewes, Lecturer on Pathology at St. Bartholomew's Hospital. Fifth Edition. (London: J. and A. Churchill. 1907. Cr. 8vo. Pp. 632. 10s. 6d.)

The fact that five editions of this book have now appeared is sufficient evidence of its popularity. In order to keep pace with the progress of pathology, the present edition, edited with the assistance of Dr. F. W. Andrewes, has been considerably enlarged, and is brought up to date by the inclusion of much that is new. Thus the chapter on tubercle has been extended to include a description of the opsonic treatment, as well as an excellent account of the manner in which tubercle bacilli spread throughout the body. The chapter on diseases of the prostate has been almost entirely rewritten, and now contains a detailed description of the adenomatous enlargement of the gland, incorrectly termed hypertrophy, whilst the constitution of the so-called "capsule" is discussed at length. Reference is also made to recent work on spirochæta pallida, the bacteriology of peritonitis, inflammatory leucocytosis, and several other points. The introductory chapters on inflammation and its sequels contain some of the best material in the book. This subject, which is often presented in such a dry and uninteresting way, is here concisely and clearly put, the general principles are carefully explained, and the student is left to apply these to the particular instance. We are surprised to find no mention of new growths of the gall-bladder, liver, or pancreas. Carcinoma of the gall-bladder is of importance on account of its very frequent association with gall-stones, and for this reason alone deserves notice. Prolapse of the rectum is attributed to the time-honoured causes—piles, calculus, polypus, or the like. We were under the impression that these were now discredited, save as quite secondary factors in the causation of rectal polypus. The absorption of the fat of the pelvic cellular tissue, which normally forms a strong supporting sheath for the rectum, readily occurs in ill-nourished individuals; and if, in addition, there is anything to cause straining, especially diarrhœa in children, prolapse is very likely to ensue. The illustrations are up to the usual standard found in text-books. This is not very high, but one with which we must be content in books of moderate price. Least satisfactory are the representations of tumours and other microscopical preparations. We have always found that those inexperienced in pathology gain a clearer idea of the histology of new growths from semi-diagrammatic drawings on a large scale than from the more accurate micro-photographs and camera-lucida drawings; and we think this plan might with advantage be introduced into the smaller text-books from which elaborate illustrations are debarred on the score of expense. This is one of the few text-books on pathology that find a place in the average student's library, and we have based our criticism on the supposition that the book is intended primarily for students. This intention, within certain limits, is excellently fulfilled.

### SPECIAL SUBJECTS.

The Ophthalmoscope: A Manual for Students. By Gustavus Hartridge, F.R.C.S. With sixty-eight illustrations and four plates. Fifth Edition. (London: J. and A. Churchill. 1907. Pp. xiii. + 153. Price 4s. net.)

The value of Mr. Hartridge's well-known manual is forcibly attested by the fact that in the fifteen years which have elapsed since its first appearance no fewer than five editions have been issued. In spite of numerous competitors it maintains its popularity, and, without question, the verdict of the student world, in this instance at least, is a just one. Doubtless the constituency interested in a book of this order is an increasing one, as recognition is now generally extended to the large clinical range open to the ophthalmoscope outside the limits of ophthalmic surgery. It is certainly to be hoped that no teacher of medicine at the present day neglects to emphasise, both by precept and example, the essential importance to the physician of a regular and systematic examination of the fundus oculi. In doing so he cannot commend to his pupils more reliable and helpful guidance than is to be found in Mr. Hartridge's manual. A similar remark may be made to those practitioners who cultivate the ambition to make good any defect in their early training by a present study of the ophthalmoscope and of the information which may be gained by its use. For the book is much more than a description of the ophthalmoscope and of the method of employing it. It is, in addition, a clinical notebook of the changes to be noted in the various parts of the eyeball as a result of disease, and of the diagnostic significance and value of these. There is no pretence to make the book a systematic treatise on diseases of the eye, but its value to the clinical student can hardly be exaggerated. Towards this result the excellent diagrams and illustrations make a substantial contribution, and, above all, the text is singularly lucid and the main purpose of the volume is kept steadily in view. We have no doubt that the book has before it a widening field of usefulness and success.

Aids to Pathology. By Harry Campbell, M.D., B.S., F.R.C.P., Senior Physician, late Pathologist, to the North-West London Hospital, Physician to the West End Hospital for Nervous Diseases. (London: Baillière, Tindall and Cox. 1908. Pp. 176. Fscp. 8vo. 10 diagrams. Price: cloth, 3s. 6d. net; paper, 3s. net.)

This little book is to all intents and purposes a cram-book. It is therefore likely to have a large sale amongst students going up for examinations. It is not a book from which pathology could be learned, nor is it one to which the practitioner would be likely to refer when he is puzzled by an obscure case. There is, it is true, a concise chapter upon the present position of opsonin and vaccine work which might be useful to many; but the rest of the book consists of short summaries of the known points in the pathology of most of the commoner conditions. There are not a few statements that are more than open to question, but it is needless to refer to these in detail when the author himself says, "There is no pretence to a complete or exhaustive treatment of the subject. The student must regard the description of each disease as a framework into which he can fit all the information acquired from the post-mortem room, the museum, and the laboratory."

